# Advanced Lymphoblastic Lymphoma, NOS in Leukaemic Phase Presenting With Sudden and Fatal CNS Haemorrhage

**DOI:** 10.1155/crip/5845893

**Published:** 2026-03-08

**Authors:** Emmanuel Kissiedu Antiri, Richard Kwasi Gyasi, Kofi Ulzen-Appiah

**Affiliations:** ^1^ Department of Haematology, Cape Coast Teaching Hospital, Cape Coast, Ghana, ccthghana.org; ^2^ Department of Haematology, University of Cape Coast School of Medical Sciences, Cape Coast, Ghana, ucc.edu.gh; ^3^ Department of Pathology, Korle-Bu Teaching Hospital, Accra, Ghana, kbth.gov.gh; ^4^ Department of Pathology, University of Ghana Medical School, Accra, Ghana, ug.edu.gh; ^5^ Department of Pathology, Cape Coast Teaching Hospital, Cape Coast, Ghana, ccthghana.org; ^6^ Department of Pathology, University of Cape Coast School of Medical Sciences, Cape Coast, Ghana, ucc.edu.gh

## Abstract

B/T‐lymphoblastic lymphoma (LBL), not otherwise specified (NOS), is a rare precursor lymphoid neoplasm that exists along a biological spectrum with acute lymphoblastic leukaemia (ALL). Although traditionally distinguished by the extent of bone marrow involvement, this separation becomes increasingly blurred in advanced or leukaemic presentations. Central nervous system (CNS) haemorrhage is a recognised but uncommon complication of acute leukaemia and is exceptionally rare as an initial manifestation within the LBL–ALL spectrum. We report a 58‐year‐old man with a 6‐month history of recurrent constitutional symptoms and anaemia, partially relieved by supportive therapy, a clinical course more suggestive of an LBL than de novo ALL. On admission, he was febrile, anaemic, thrombocytopenic and hypoxic, with hepatomegaly and mild lymphadenopathy, and deteriorated rapidly, dying within 24 h. Post‐mortem examination revealed a hypercellular bone marrow extensively replaced by lymphoblasts, widespread multiorgan infiltration and a large intracerebral haemorrhage with ventricular and subarachnoid extension. Histological examination demonstrated leukostasis, perivascular lymphoblast infiltration, leukaemic nodules and areas of both acute and chronic haemorrhage. The chronic clinical course, limited nodal disease and extensive marrow and extranodal involvement support an interpretation of advanced LBL, NOS in the leukaemic phase. This presentation also highlights the substantial biological overlap with ALL. This case highlights the risk of catastrophic CNS haemorrhage across the LBL–ALL spectrum and reinforces the need for vigilance, timely diagnostics and the enduring value of autopsy in clarifying unexpected deaths.

## 1. Introduction

B/T‐lymphoblastic leukaemia/lymphoma (LBL/L) is a precursor lymphoid neoplasm composed of small‐ to medium‐sized blast cells that show scanty cytoplasm, dispersed to moderately condensed chromatin and indistinct nucleoli [1]. The term “lymphoma” is applied when the disease is confined to a mass lesion with minimal marrow or blood involvement, whereas “leukaemia” is used when marrow and blood infiltration predominates. In clinical practice, a threshold of greater than 25% marrow blasts is generally used to define acute lymphoblastic leukaemia (ALL) [1]. ALL and lymphoblastic lymphoma (LBL) exist along a biological spectrum [2]. In advanced or leukaemic presentations, the distinction between the two is largely based on disease distribution rather than fundamental biological differences.

While LBL most often presents with nodal or extranodal masses, progression to a leukaemic phase with extensive marrow involvement may occur, further blurring the distinction from ALL and complicating the clinical course. In this advanced phase, severe cytopenias, hyperleukocytosis with leukostasis and central nervous system (CNS) infiltration can develop.

In one report, a 7‐year‐old girl with T‐cell ALL and extreme hyperleukocytosis developed extensive punctate intracranial haemorrhages (ICHs) with cranial nerve palsies during induction therapy. With rapid initiation of chemotherapy, she achieved a full neurological recovery within 3 months, demonstrating the lifesaving potential of early recognition and treatment [3].

Another report described a 17‐year‐old male with no prior diagnosis who presented with sudden massive intracerebral and intraventricular haemorrhage, midline shift and uncal herniation. Despite emergent surgical evacuation, the patient progressed to brain death, illustrating a rare instance of fatal ICH as the first manifestation of ALL [4].

In Africa, documentation of lymphoblastic neoplasms and their CNS complications remains limited, with most publications focusing on more common adult non‐Hodgkin lymphomas such as diffuse large B‐cell lymphoma (DLBCL). Reports from sub‐Saharan Africa describe the burden and poor outcomes of acute leukaemia; however, cases occurring along the LBL–ALL spectrum presenting with rapidly fatal CNS haemorrhage are rarely detailed and likely underreported.

This report is aimed at broadening clinical awareness, highlighting diagnostic challenges and drawing attention to the possibility of sudden fatal CNS haemorrhage in advanced lymphoblastic neoplasms occurring along the LBL–ALL spectrum, particularly within resource‐limited settings.

## 2. Case Presentation

A 58‐year‐old male with no prior comorbidities presented with a 6‐month history of progressive fatigue, malaise, intermittent fever and symptoms of anaemia, for which he had received antibiotics, haematinics and two units of red cell transfusion at private clinics. A referral blood count showed anaemia (Hb 8.1 g/dL), leukocytosis (22 × 10^9^/L, predominantly lymphocytes) and thrombocytopenia (44 × 10^9^/L). He was scheduled for bone marrow aspiration and immunophenotyping, but deteriorated before the procedure.

**Figure 1:**
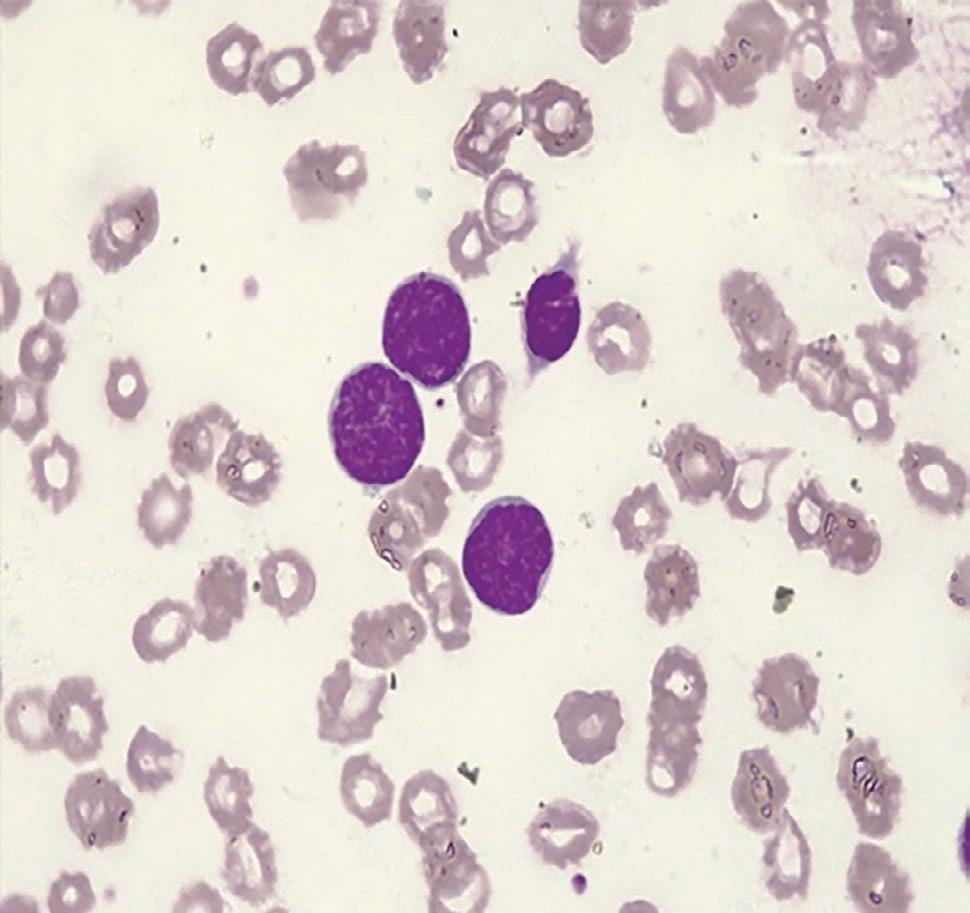
(Leishman stain ×100): Large abnormal cells with scanty cytoplasm, immature nuclear chromatin and occasional nucleolus. Platelets markedly reduced.

On admission to the emergency unit, he was acutely ill, febrile (41°C), severely pale, tachycardic, hypotensive and hypoxic, with hepatomegaly and inguinal lymphadenopathy. His GCS was 4/15 with sluggish pupils. Laboratory investigations also revealed elevated lactate dehydrogenase (LDH) and uric acid. Ultrasound confirmed hepatomegaly. Despite oxygen, fluids and supportive care, he died less than 24 h after admission. Posthumous review of his peripheral blood film supported a diagnosis of LBL, not otherwise specified (NOS) in the leukaemic phase showing 81% large abnormal cells with scanty cytoplasm, immature nuclear chromatin and occasional nucleolus (Figure 1).

## 3. Autopsy Findings

Internal examination revealed pallor of the conjunctiva, mucous membranes and serous surfaces, with lymphadenopathy (submandibular, cervical and inguinal nodes). The heart (390 g), lungs (right 610 g and left 520 g), liver (2980 g), kidneys (350 g and 280 g) and spleen (330 g) were all heavier than normal. The kidneys were pale, the lungs were congested and oedematous, and the liver showed diffuse tan lesions and petechiae on both capsular and cut surfaces. Similar petechial haemorrhages were present on the renal subcapsular surface. The spleen was enlarged and firm.

**Figure 2:**
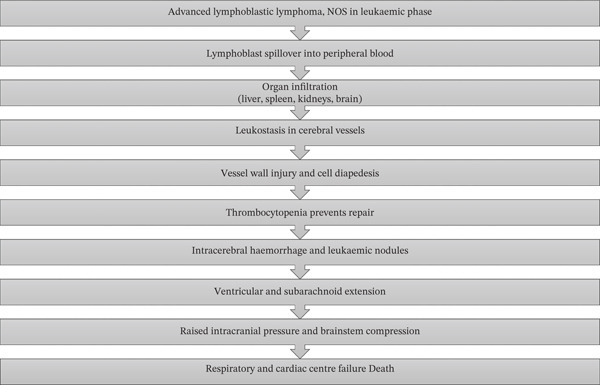
Pathophysiological cascade in advanced lymphoblastic lymphoma, not otherwise specified (NOS) in the leukaemic phase, showing progression from lymphoblast spillover and multiorgan infiltration to leukostasis, thrombocytopenia, intracerebral haemorrhage, raised intracranial pressure and fatal brainstem compression.

**Figure 3:**
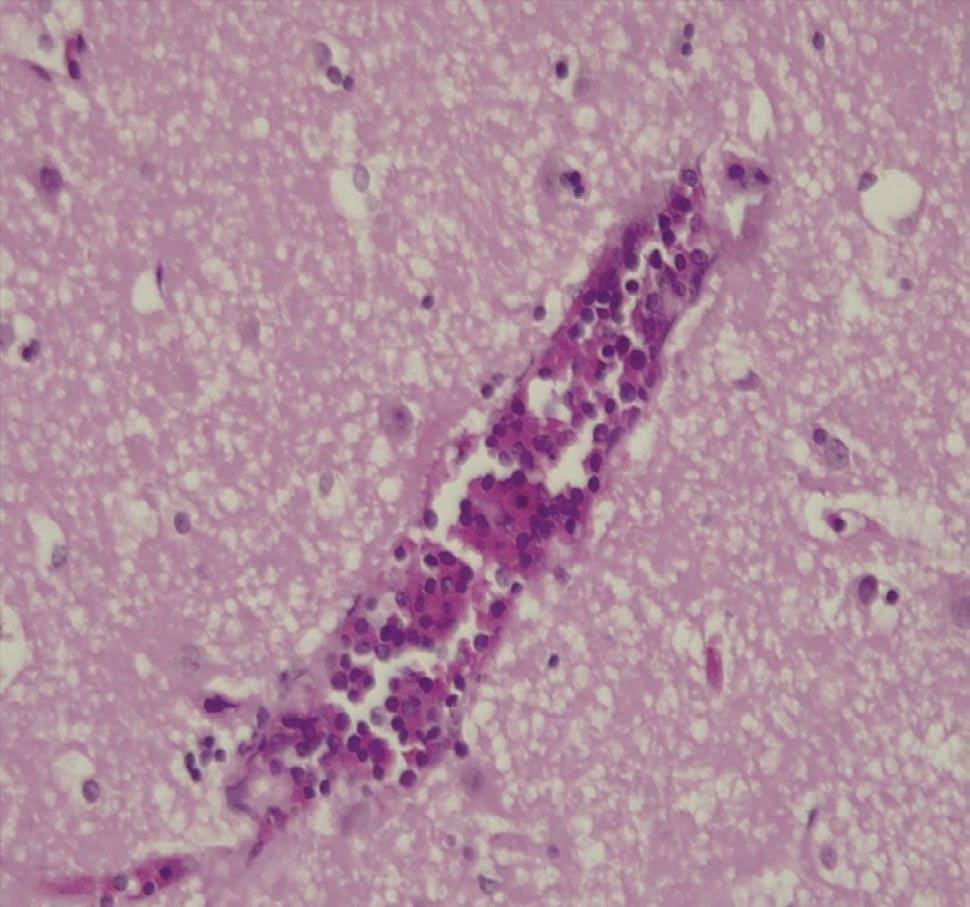
*H&E ×40.* Diffuse infiltration of the cerebral cortex with small‐ to medium‐sized lymphoblasts, some within the congested vessel lumen in the cerebrum. Leukostasis is present with the vessel lumen filled with leukaemic cells interspersed among red cells. There is vascular damage with diffuse infiltrate of leukaemic cells within the cerebral parenchyma, with the cells spreading between glial cells and around the vessel.

**Figure 4:**
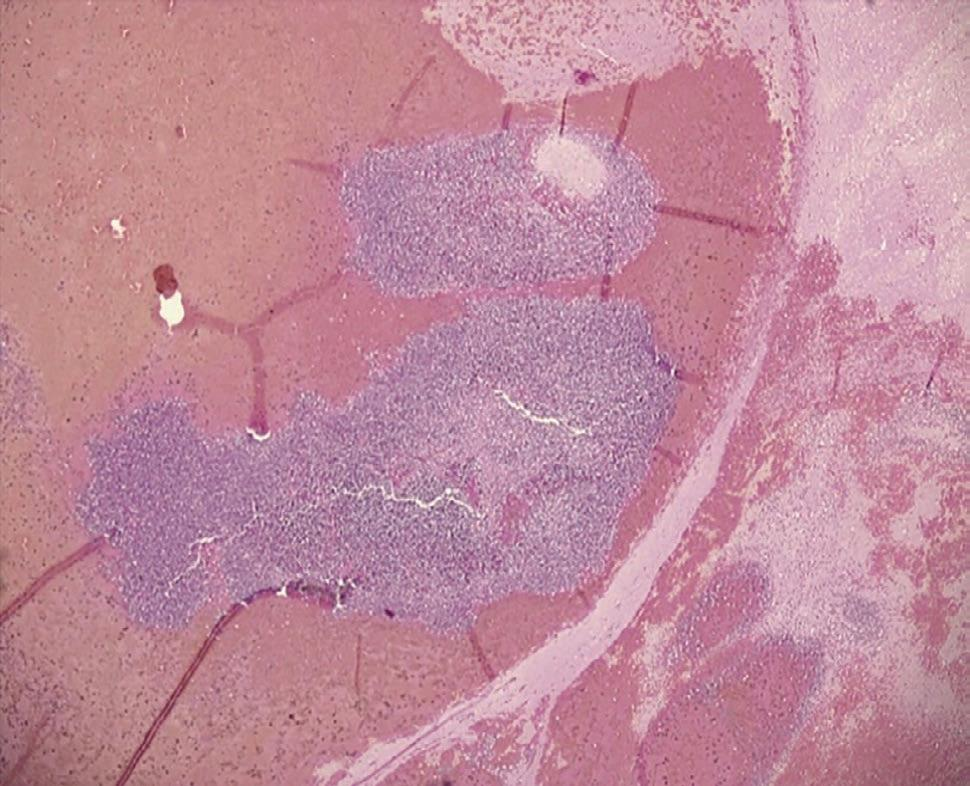
H&E *×*4. Large intracerebral parenchymal aggregates of lymphoblasts (leukaemic nodules) with evidence of haemorrhage.

The brain weighed 1200 g and showed a large left intracerebral parenchymal haemorrhage with ventricular extension and subarachnoid spread, compressing adjacent structures (thalamus, internal capsule, putamen and globus pallidus) (Figures 2, 3 and 4). Bone marrow aspiration performed at autopsy was hypercellular with markedly reduced megakaryocytes and numerous ruptured lymphocytes due to increased cellular fragility, making morphological assessment difficult.

## 4. Histopathology

Histological sections demonstrated widespread infiltration by small‐ to medium‐sized lymphoblasts with a high nuclear‐to‐cytoplasmic ratio and occasional nucleoli. Diffuse cortical and perivascular infiltration of the brain by lymphoblasts was observed, with associated vascular damage, leukaemic nodules and areas of both old and new intraparenchymal haemorrhage. The lymph node showed effacement of nodal architecture by sheets of lymphoblasts, with secondary follicles containing numerous immature lymphoid cells. The bone marrow was hypercellular and largely replaced by lymphoblasts, with residual erythroid precursors, small lymphocytes, plasma cells and a few megakaryocytes. The spleen demonstrated marked architectural effacement with diffuse lymphoblastic infiltration of red and white pulp, and vascular lumina also contained lymphoblasts. In the liver, sheets of lymphoblasts infiltrated the lobular, sinusoidal and periportal regions. The kidneys showed tubular necrosis with heavy interstitial and glomerular infiltration by lymphoblasts.

## 5. Discussion

B/T‐LBL, NOS and ALL exist along a biological spectrum, with marrow blast percentage conventionally used to separate the two. In advanced or leukaemic presentations, this distinction becomes less clear and reflects disease distribution rather than a definitive biological separation. In our patient, an interpretation of advanced LBL, NOS in the leukaemic phase was supported by a 6‐month chronic course with partial symptomatic improvement and relapse, which is uncharacteristic of de novo ALL. This was further supported by the presence of only a few mildly enlarged lymph nodes rather than the more generalised lymphadenopathy typically seen in ALL. Extensive marrow infiltration with 81% circulating blasts (Figure 1), together with infiltration of multiple organs, confirmed advanced disease along the LBL–ALL spectrum.

The following pathophysiological sequence is inferred from gross pathological and histological findings at autopsy and is grounded in established principles of haematopathology [5–7]. However, the precise temporal sequence and causal hierarchy of these mechanisms in this individual case cannot be definitively established.

**Figure 5:**
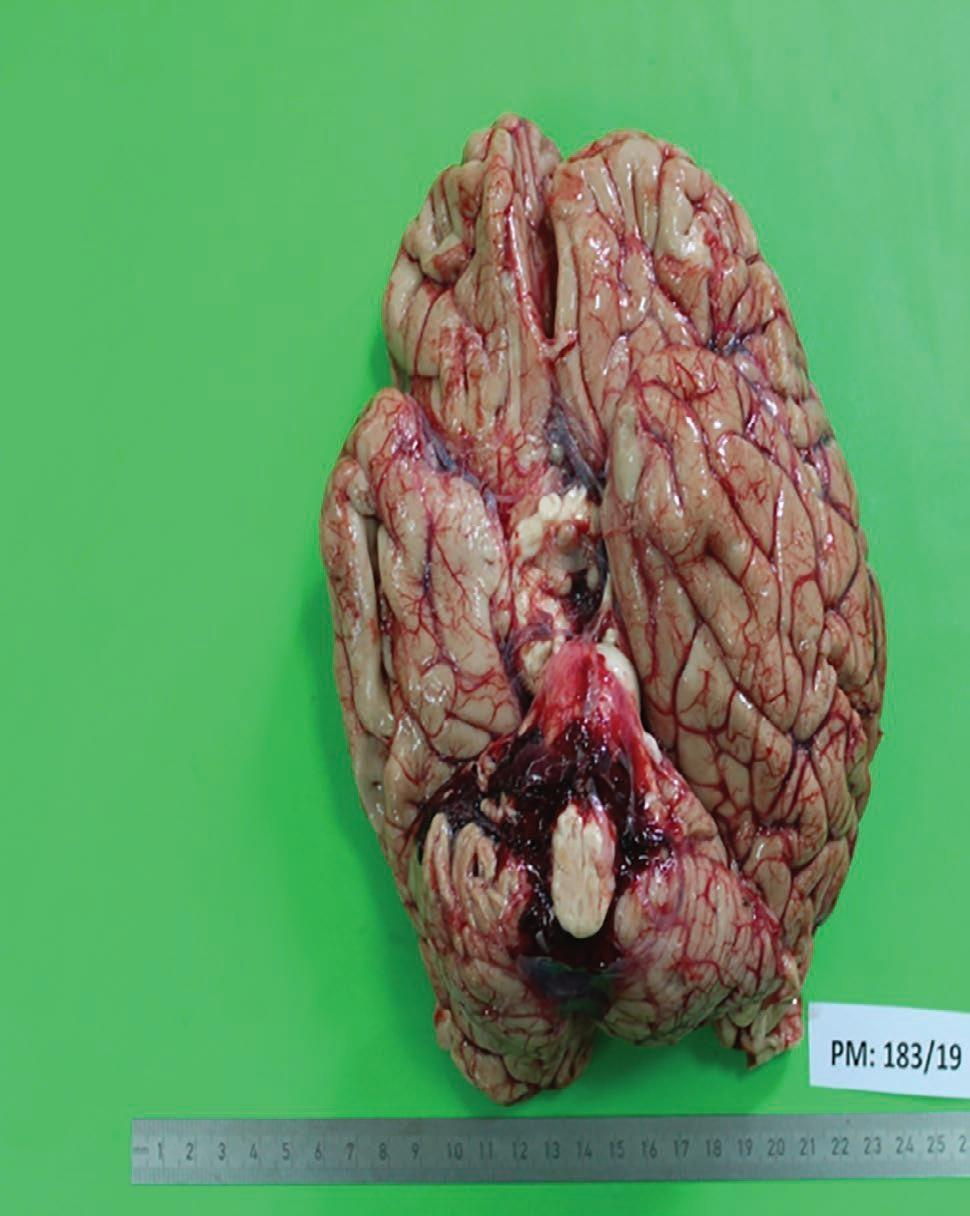
Haemorrhage seen around the brain stem upon examining the base of the brain, likely an intracerebral parenchymal haemorrhage with subarachnoid extension.

**Figure 6:**
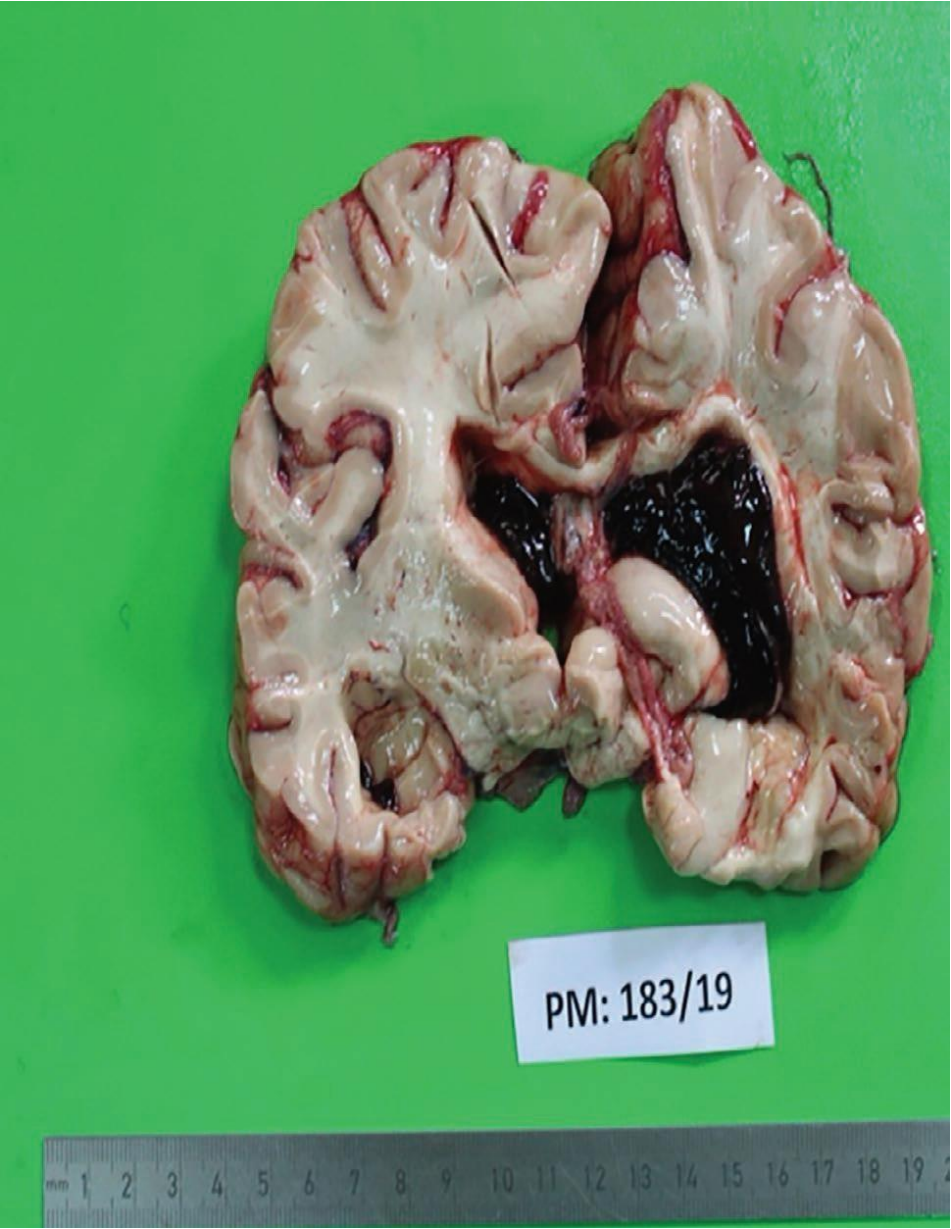
Cut surface of the cerebrum showing left intracerebral parenchymal haemorrhage with ventricular extension. There is compression of surrounding structures by the haemorrhage. There is ventricular system dilatation with haemorrhage marked in the left lateral ventricle.

**Figure 7:**
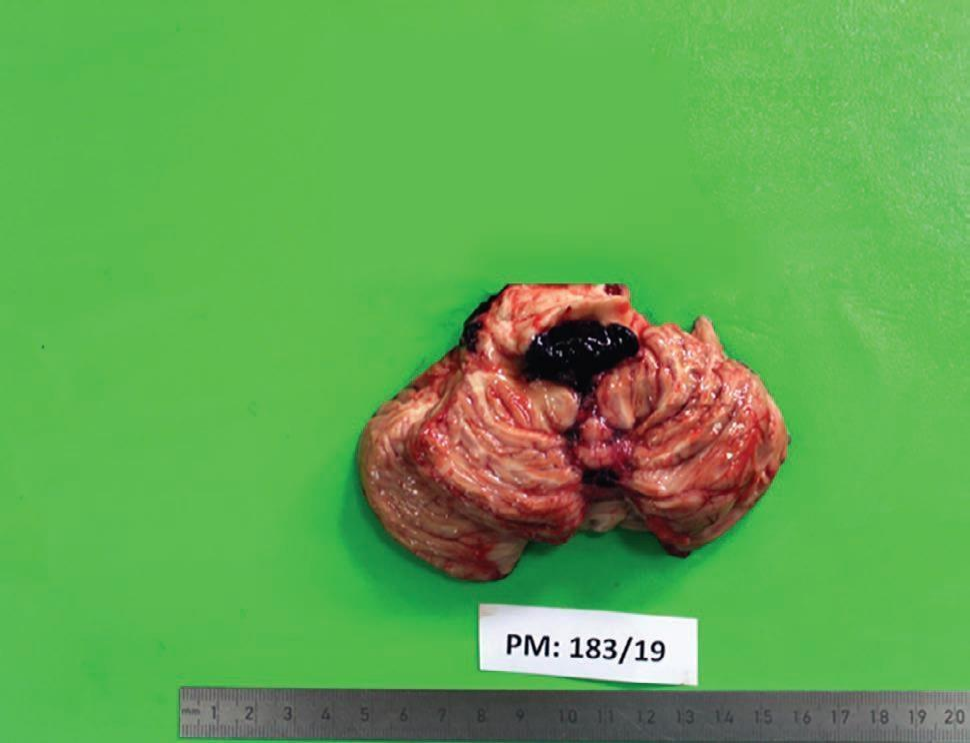
Pathophysiological cascade in advanced lymphoblastic lymphoma, not otherwise specified (NOS) in the leukaemic phase, showing progression from lymphoblast spillover and multiorgan infiltration to leukostasis, thrombocytopenia, intracerebral haemorrhage, raised intracranial pressure and fatal brainstem compression.

The sequence of events (Figure 2) culminating in death likely began with lymphoblasts spilling into the peripheral blood and infiltrating the liver, spleen, kidneys and brain. Within the brain, heavy infiltration of the cerebral hemispheres was observed, likely facilitated by leukostasis in elongated white matter vessels that are particularly vulnerable to thrombus formation (Figure 3). Vascular stasis likely impaired microvascular nutrition and increased permeability, thereby facilitating lymphoblast diapedesis into the parenchyma (Figure 3). Severe thrombocytopenia likely compromised effective vascular repair and predisposed to widespread haemorrhage. Expansion of Virchow–Robin spaces by cells and proteinaceous fluid was associated with the formation of leukaemic nodules. These nodules compressed small capillaries and contributed to fresh haemorrhage around older foci (Figure 4). The catastrophic left intracerebral haemorrhage extended into the ventricular system and subarachnoid space. It was associated with raised intracranial pressure, compression of the thalamus and brainstem, and ultimately fatal cardiorespiratory compromise (Figures 5, 6 and 7).

While leukostasis, vascular infiltration by lymphoblasts, thrombocytopenia and intracerebral haemorrhage were directly demonstrated at gross and histological examination, the temporal sequence and relative contribution of these processes are inferred and should be interpreted with appropriate caution.

Comparison with previously published cases illustrates the spectrum of outcomes associated with lymphoblastic neoplasms complicated by CNS haemorrhage. A child with T‐cell ALL and hyperleukocytosis survived extensive punctate ICHs following rapid initiation of chemotherapy [3]. In contrast, a previously undiagnosed adolescent with ALL succumbed to massive intracerebral haemorrhage despite surgical intervention [4]. Our patient followed the latter trajectory. Late presentation, profound marrow failure, incomplete disease classification due to limited immunophenotyping and the absence of disease‐directed therapy culminated in rapid clinical deterioration and death. The immediate cause of death was a left intracerebral parenchymal haemorrhage with subarachnoid extension, resulting from severe thrombocytopenia secondary to advanced LBL, NOS in the leukaemic phase. This case underscores the importance of early recognition, adequate diagnostic capacity and the continued relevance of autopsy in elucidating unexpected clinical outcomes, particularly in resource‐limited settings.

Under the WHO Classification of Haematolymphoid Tumours, 5th edition, lymphoid neoplasms are classified using a hierarchical framework that progresses from broad disease categories to increasingly specific entities and subtypes [8]. In circumstances where comprehensive immunophenotypic or molecular characterisation is not feasible because of limited resources or tissue availability, assignment at a higher diagnostic level is considered appropriate [8]. In the present case, the absence of immunophenotypic and genetic data precluded definitive lineage assignment. Therefore, the diagnosis was retained at the family/class level as LBL, NOS in the leukaemic phase. Because lineage could not be definitively established, comparisons with previously reported lineage‐defined ALL or LBL cases should be interpreted cautiously and are intended to highlight shared clinical behaviour and catastrophic CNS complications across the LBL–ALL spectrum, rather than to imply diagnostic equivalence.

## 6. Limitation

A major limitation of this case was the inability to perform bone marrow aspiration and immunophenotypic studies while the patient was alive, investigations that are essential for confirming lineage (B‐cell vs. T‐cell) and precise subclassification of lymphoblastic neoplasms. In the absence of these data, the diagnosis was necessarily restricted to LBL, NOS in the leukaemic phase rather than a lineage‐defined B‐ or T‐LBL.

In addition, given the recognised biological overlap between LBL and ALL in advanced or leukaemic presentations, the diagnostic classification in this case should be interpreted within the context of this disease spectrum rather than as a rigid categorical distinction. In line with the current WHO Classification of Haematolymphoid Tumours (5th edition) [8], further genetic and molecular subclassification is recommended; however, such studies could not be performed either ante‐mortem or post‐mortem, as immunophenotypic and molecular testing was not readily available at the time of autopsy in 2019, and archived tissue is no longer available for additional analysis during manuscript revision.

## 7. Conclusion

This case illustrates an aggressive presentation of advanced LBL, NOS in the leukaemic phase, with diffuse multiorgan infiltration and fatal outcome within 24 h of hospital admission. It highlights the clinical and pathological overlap between LBL and ALL, now recognised as closely related entities along a single disease spectrum in the current WHO classification. Early recognition, accurate immunophenotypic characterisation and timely initiation of therapy remain essential for improving survival in such patients. Importantly, this case also highlights the underestimated risk of catastrophic CNS haemorrhage in lymphoblastic neoplasms occurring along the LBL–ALL spectrum, which can lead to sudden and fatal deterioration even before treatment is initiated.

## Funding

No funding was received for this manuscript.

## Consent

Written informed consent was obtained from the patient′s next of kin for publication of this case report and all associated clinical and pathological images. A copy of the written consent is available for review by the journal editor upon request.

## Conflicts of Interest

The authors declare no conflicts of interest.

## Data Availability

The data that support the findings of this study are available from the corresponding author upon reasonable request.
