# Correction to ‘Advanced Lymphoblastic Lymphoma, NOS in Leukaemic Phase Presenting With Sudden and Fatal CNS Haemorrhage’

**DOI:** 10.1155/crip/9856983

**Published:** 2026-09-28

E. K. Antiri, R. K. Gyasi, and K. Ulzen‐Appiah, ‘Advanced Lymphoblastic Lymphoma, NOS in Leukaemic Phase Presenting With Sudden and Fatal CNS Haemorrhage,’ Case Reports in Pathology 2026, no. 1 (2026): 1–6, 10.1155/crip/5845893.

In the article titled ‘Advanced Lymphoblastic Lymphoma, NOS in Leukaemic Phase Presenting With Sudden and Fatal CNS Haemorrhage’, there were errors in the order of Figures 2, 3, 4, 5, 6 and 7. These errors were introduced by the publisher during the production of the article and are as follows:

The image published as Figure 2 should be Figure 7.

The image published as Figure 3 should be Figure 5.

The image published as Figure 4 should be Figure 6.

The image published as Figure 5 should be Figure 2.

The image published as Figure 6 should be Figure 3.

The image published as Figure 7 should be Figure 4.

Additionally, the legend presented within the image published as Figure 2 was mistakenly duplicated in Figure 7. This occurred due to the error in figure ordering.

The figures as the authors intended are shown below:

**Figure 2:**
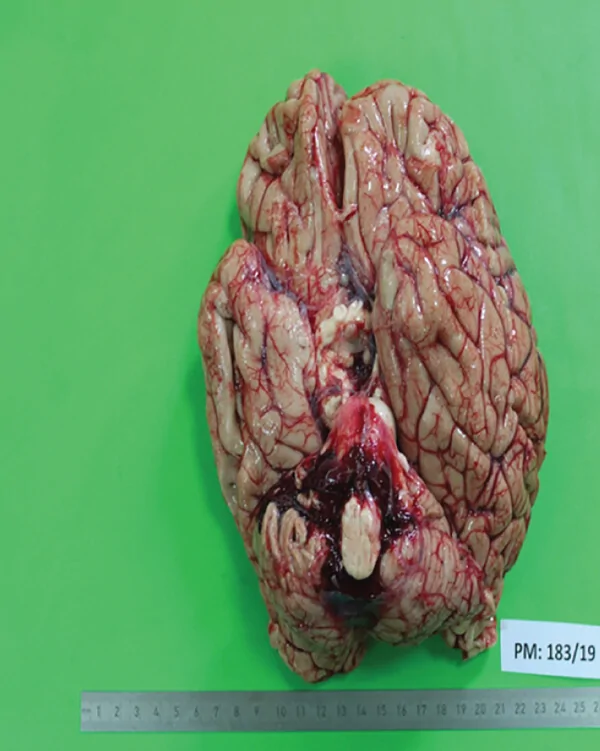
Haemorrhage seen around the brain stem upon examining the base of the brain, likely an intracerebral parenchymal haemorrhage with subarachnoid extension.

**Figure 3:**
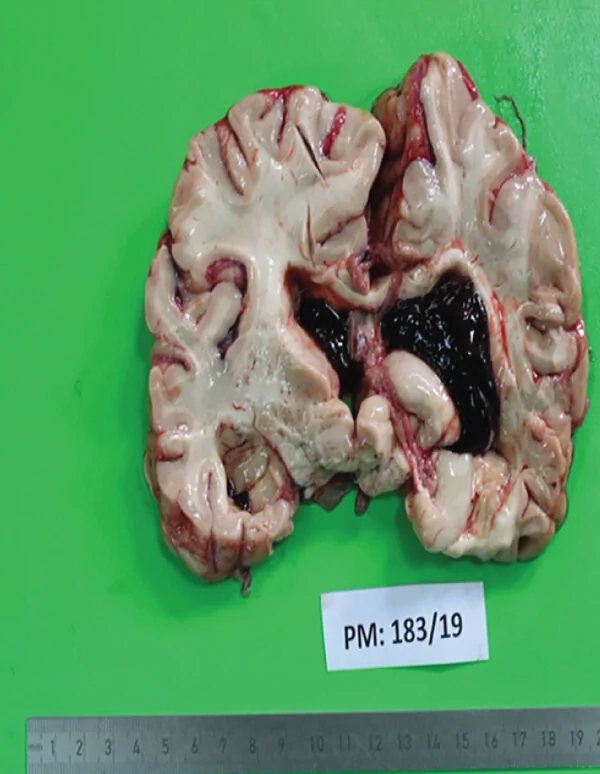
Cut surface of the cerebrum showing left intracerebral parenchymal haemorrhage with ventricular extension. There is compression of surrounding structures by the haemorrhage. There is ventricular system dilatation with haemorrhage marked in the left lateral ventricle.

**Figure 4:**
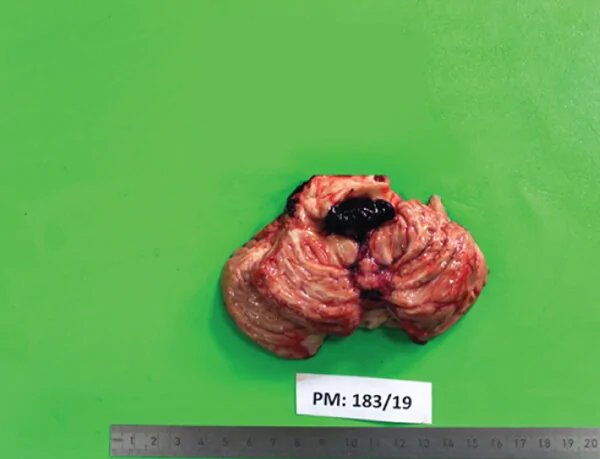
Cut surface of the brain stem shows an intraventricular haemorrhage.

**Figure 5:**
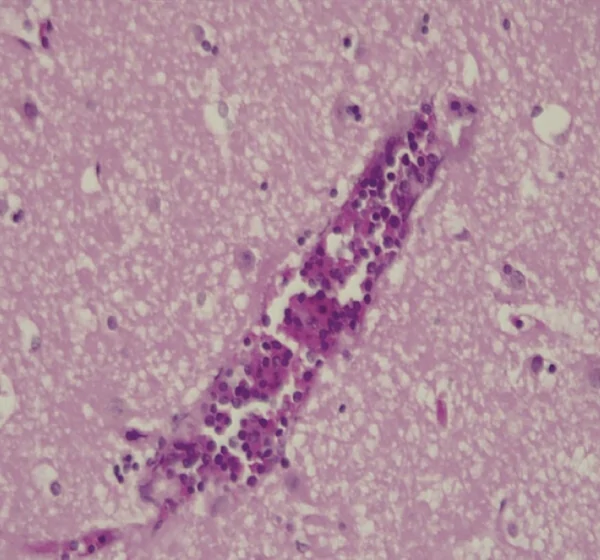
H&E ×40. Diffuse infiltration of the cerebral cortex with small‐ to medium‐sized lymphoblasts, some within the congested vessel lumen in the cerebrum. Leukostasis is present with the vessel lumen filled with leukaemic cells interspersed among red cells. There is vascular damage with diffuse infiltrate of leukaemic cells within the cerebral parenchyma, with the cells spreading between glial cells and around the vessel.

**Figure 6:**
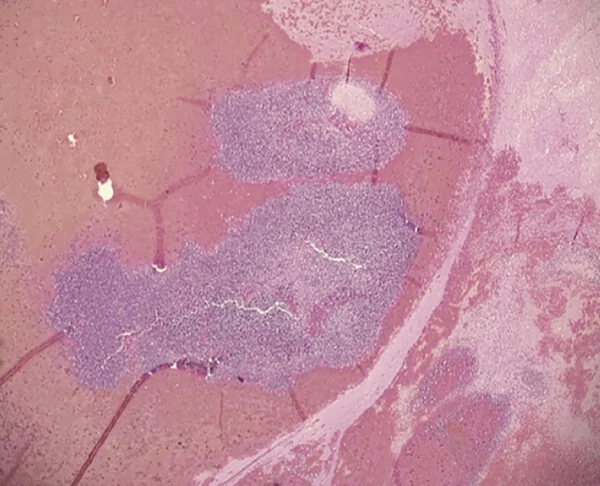
H&E ×4. Large intracerebral parenchymal aggregates of lymphoblasts (leukaemic nodules) with evidence of haemorrhage.

**Figure 7:**
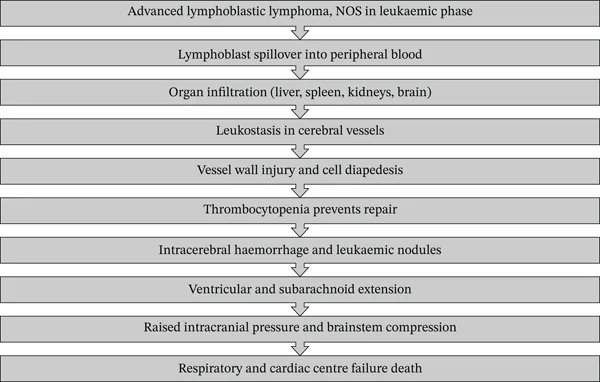
Pathophysiological cascade in advanced lymphoblastic lymphoma, not otherwise specified (NOS) in the leukaemic phase, showing progression from lymphoblast spillover and multiorgan infiltration to leukostasis, thrombocytopenia, intracerebral haemorrhage, raised intracranial pressure and fatal brainstem compression.

We apologize for these errors.

